# Rise and Fall of an Anti-MUC1 Specific Antibody

**DOI:** 10.1371/journal.pone.0015921

**Published:** 2011-01-14

**Authors:** Holger Thie, Lars Toleikis, Jiandong Li, Reinhard von Wasielewski, Gunther Bastert, Thomas Schirrmann, Isabel Tourais Esteves, Christian K. Behrens, Bénédict Fournes, Nathalie Fournier, Christophe de Romeuf, Michael Hust, Stefan Dübel

**Affiliations:** 1 Technische Universität Braunschweig, Institut für Biochemie und Biotechnologie, Braunschweig, Germany; 2 Hannover Medical School, Institute of Pathology, Hannover, Germany; 3 Universitätsfrauenklinik Heidelberg, Heidelberg, Germany; 4 LFB Biotechnologies - Research Department, Lille, France; National Jewish Health, United States of America

## Abstract

**Background:**

So far, human antibodies with good affinity and specificity for MUC1, a transmembrane protein overexpressed on breast cancers and ovarian carcinomas, and thus a promising target for therapy, were very difficult to generate.

**Results:**

A human scFv antibody was isolated from an immune library derived from breast cancer patients immunised with MUC1. The anti-MUC1 scFv reacted with tumour cells in more than 80% of 228 tissue sections of mamma carcinoma samples, while showing very low reactivity with a large panel of non-tumour tissues. By mutagenesis and phage display, affinity of scFvs was increased up to 500fold to 5,7×10^−10^ M. Half-life in serum was improved from below 1 day to more than 4 weeks and was correlated with the dimerisation tendency of the individual scFvs. The scFv bound to T47D and MCF-7 mammalian cancer cell lines were recloned into the scFv-Fc and IgG format resulting in decrease of affinity of one binder. The IgG variants with the highest affinity were tested in mouse xenograft models using MCF-7 and OVCAR tumour cells. However, the experiments showed no significant decrease in tumour growth or increase in the survival rates. To study the reasons for the failure of the xenograft experiments, ADCC was analysed *in vitro* using MCF-7 and OVCAR3 target cells, revealing a low ADCC, possibly due to internalisation, as detected for MCF-7 cells.

**Conclusions:**

Antibody phage display starting with immune libraries and followed by affinity maturation is a powerful strategy to generate high affinity human antibodies to difficult targets, in this case shown by the creation of a highly specific antibody with subnanomolar affinity to a very small epitope consisting of four amino acids. Despite these “best in class” binding parameters, the therapeutic success of this antibody was prevented by the target biology.

## Introduction

*In vitro* selection by phage display is a powerful and proven technology to generate antibodies [1]–[4] against nearly any target [5]–[7], including toxins [8]–[10], pathogens [11]–[13] or haptens [14]. It has yielded therapeutic antibodies [15], [16] and binders with properties superior to conventional (animal based) methods, and of human origin (for review see [6], [17]). Phage dispay, after a mutagenesis strategy, further allows to improve the biochemical properties of antibodies, for example for affinity maturation [18], [19]. The availability of these methods has thoroughly affected the validation of antibodies for therapeutic strategies, recognising a very high affinity as a substantial property of any lead candidate. However, the generation of very high affinity antibodies has proven to be difficult to some promising tumour targets, thus substantially hindering their use for cancer treatment. Despite that, novel cancer treatment strategies became possible by using recombinant antibodies. One blockbuster example is trastuzumab (Herceptin®), a humanised anti-Her2 antibody used in breast cancer treatment. This antibody blocks the overexpression of Her2 receptor which is responsible for an aggressive disease progression combined with a poor prognosis [20]. However, since Her2 is overexpressed only in around 20% of all breast tumours, other tumour antigens would be urgently needed for antibody-based cancer therapies. One possible antigen is MUC1 (also known as CD227, PUM or CA-15-3). MUC1 is overexpressed on 90% of breast cancers [21], [22] and other cancers, e.g. prostate cancer [23], [24]. It is a heavily O-glycosylated transmembrane protein, which is found on the luminal surface of many epithelial cells in duct tissue [25], [26]. MUC1 has a molecular mass of more than 400 kDa [27] and consists of three domains, a 69 amino acid cytoplasmatic domain involved in several signaling processes [28]–[30], a transmembrane domain of 31 amino acids [31] and a very large exo-domain, which is responsible for most of the molecular mass. This domain consists mainly of a repetitive 20 amino acid sequence, which is termed VNTR (variable number of tandem repeats) in homology to its corresponding genetic structure [32], [33], [31], [34]. The number of repeats in the VNTR domain varies between 20 and 120, with 40–80 typically found in MUC1 [33], [31], [35], [36]. Two serine and three threonine residues are found per repeat. The hydroxyl groups of these amino acid residues are potential O-glycosylation sites [27] which finally results in an oligosaccharide content of more than 50% of the molecular mass of MUC1 [31]. The O-glycosylations found in MUC1 of normal epithelial tissue consist of long and branched sugar structures from the polyactosamine type containing typically 8–10 monosaccharide units [37]–[39]. This highly glycosylated MUC1 binds water, leading to a moisturisation of the cell surface. It protects the cell from proteolytic attacks, avoids the colonisation by microorganisms [40], [41] and regulates cell-cell and cell-extracellular matrix interactions [42], [43]. In tumour cells, the apical expression of MUC1 is lost and the apolar expression leads to MUC1 presentation over the entire cell surface [44] resulting in an accessibility by systematically administered antibodies [45]. Some tumour associated MUC1 is sheded into the circulation [46], [47]. Most significantly, the O-glycosylation patterns found in the VNTRs are different between tumour MUC1 and MUC1 expressed by normal epithelial cells. Instead of long and branched sugar chains, less complex and shorter glycosylation patterns are found in tumours [35], [37], [38], [48], [49]. These differences lead to the presentation of new epitopes on the surface of tumour cells mainly by exposing the formerly masked peptide backbone of MUC1 to antibodies [50]. Hence, the different MUC1 properties allow to discriminate and to attack MUC1 positive tumour cells using antibodies specific to these cancer specific neo-epitopes.

Several antibodies against MUC1 were developed over the last thirty years. One of the most prominent is the murine antibody HMFG1, which was first published in 1981 [44]. This antibody recognizes a peptide epitope (PDTR) within the VNTR region of the extracellular domain of MUC1. It was humanized [51] and afterwards clinically developed by Antisoma for the treatment of breast cancer (huHMFG1/AS1402/R1550/Therex). Another humanised anti-MUC1 antibody (GT-MAB 2.5-GEX, formerly PankoMab) [45], [52] recognizing a glycosylated PDTR motif is currently in a clinical phase I study.

A large panel of rodent MUC1 antibodies was investigated in 1998 in a MUC1 workshop [53] showing that most antibodies have different fine specificities and affinities in a range from KD  = 3–400 nM. In contrast, human antibodies were found to have only low affinities [54], [55].

In this study, we describe the selection of a human anti-MUC1 scFv antibody derived from a phage display immune antibody gene library of breast cancer patients repeatedly vaccinated with synthetic MUC1 glycopeptides. The selected binder was affinity and stability-matured by random mutagenesis and phage display selection, biochemically characterised, and analysed *in vitro* and *in vivo* for anti-cancer activity.

## Results

### Vaccination of breast cancer patients

Six breast cancer patients were repeatedly vaccinated with a synthetic MUC1 glycopeptide. All patients received the synthetic 15mer MUC1-glycopeptide (APDT(GalNAc)RPAPGSTAPPA) conjugated to KLH (Keyhole limpet hemocyanin). A total of four to eight vaccinations per patient were administered every week and IgG and IgM antibody production against MUC1 glycopeptide, purified MUC1 (Breast Mucin Antigen, BMA) and KLH was analysed by ELISA using sera of vaccinated patients (S. Kaul, Universitätsfrauenklinik Heidelberg, unpublished data). All patients developed a significant increase in both IgG and IgM titres after vaccination. The induced serum antibodies showed strong binding to synthetic peptides and glycopeptides representing the VNTR region of MUC1 as well as to KLH. These antibodies did not show any reactivity to whole human MUC1 protein, which was purified by affinity chromatography from cells of the MUC1 overexpressing breast cancer cell line T47D.

### Construction of a human antibody gene library from blood of vaccinated breast cancer patients

Peripheral B cells were isolated from six breast cancer patients vaccinated with synthetic MUC1 glycopeptide. The mRNA was extracted and reverse transcribed into cDNA. The cDNA was pooled and used as template for PCR amplification of VH and VL genes with a specific set of oligonucleotide primers [56]. The human scFv library was cloned in two steps. In the first step, the pool of VH genes, amplified with IgG specific primers, were cloned into the phagemid pSEX81 [57]. In the second step, the VL gene segments were cloned into pSEX81 containing the VH gene repertoire. The obtained diversities were 6.5×10^5^ individual clones for the kappa sublibrary and 1.7×10^6^ individual clones for the lambda sublibrary, respectively.

### Selection of human anti-MUC1 scFv

**Figure 1:**
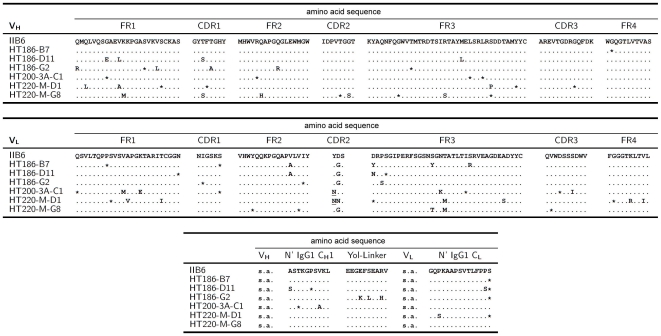
Comparison of scFv amino acid sequences of IIB6 and affinity matured anti-MUC1 scFvs. Differences in amino acid sequences are given by the corresponding amino acid in the table (single letter code). * represents a silent point mutation leading to no change in amino acid sequence. The upper panel shows the VH alignment, the middle pannel shows the VL alignment and the bottom pannel shows the alignment of the linker sequences between VH and VL, consisting of the N-terminal part of CH1 and the yol epitope, and the N-terminal part of CL downstream of VL. s.a.  =  see above.

Three panning rounds were performed on purified MUC1 (BMA), followed by one panning round on synthetic MUC1 glycopeptide. Forty-two out of 46 analysed clones showed the desired binding specificity to purified MUC1 (BMA), synthetic MUC1 glycopeptides and to a cell lysate of T47D cells, whereas control antigens were not bound (data not shown). The phagemid DNA of the 42 individual clones was prepared and sequencing revealed that all 42 clones were identical. Clone IIB6 was used for further studies. The variable domain of IIB6 heavy chain was derived from IGHV1-2, IGHD3-10 and IGHJ4 germline gene fragments as determined by VBASE2 (www.vbase2.org) [58]. The lambda light chain was derived from IGLV3-21 and IGLJ3 germline gene fragments (Fig. 1).

### Specificity, affinity and stability analysis of human anti-MUC1 scFv

**Figure 2:**
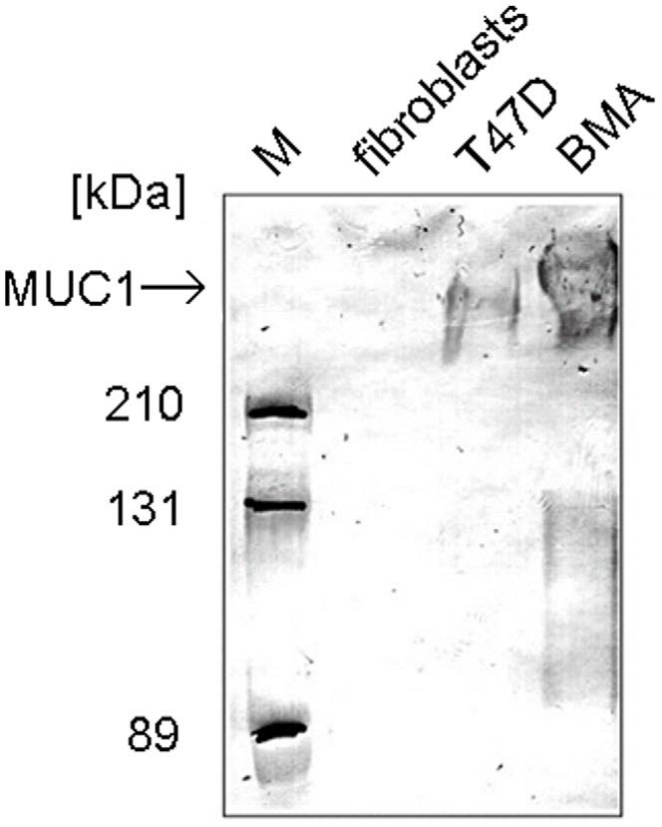
IIB6 binding to MUC1. MUC1 negative fibroblast, MUC1 positive T47D cells and MUC1 preparations (BMA) were separated by 7.5% SDS-PAGE and Western-blotted. The blot was stained with 5 µg/ml IIB6, mouse anti-his tag (1∶1000) and goat anti-mouse IgG (Fab specific) HRP conjugate (1∶5000)

The specificity of the scFv fragment IIB6 was analysed by various assays. The specific binding of the scFv antibody to the VNTR region of tumour-associated MUC1 was confirmed by ELISA (data not shown) and immunoblot (Fig. 2). Purified MUC1 as well as a cell lysate of T47D cells were bound by scFv IIB6 determined by immunoblot whereas a cell lysate of fibroblasts was not bound.

**Figure 3:**
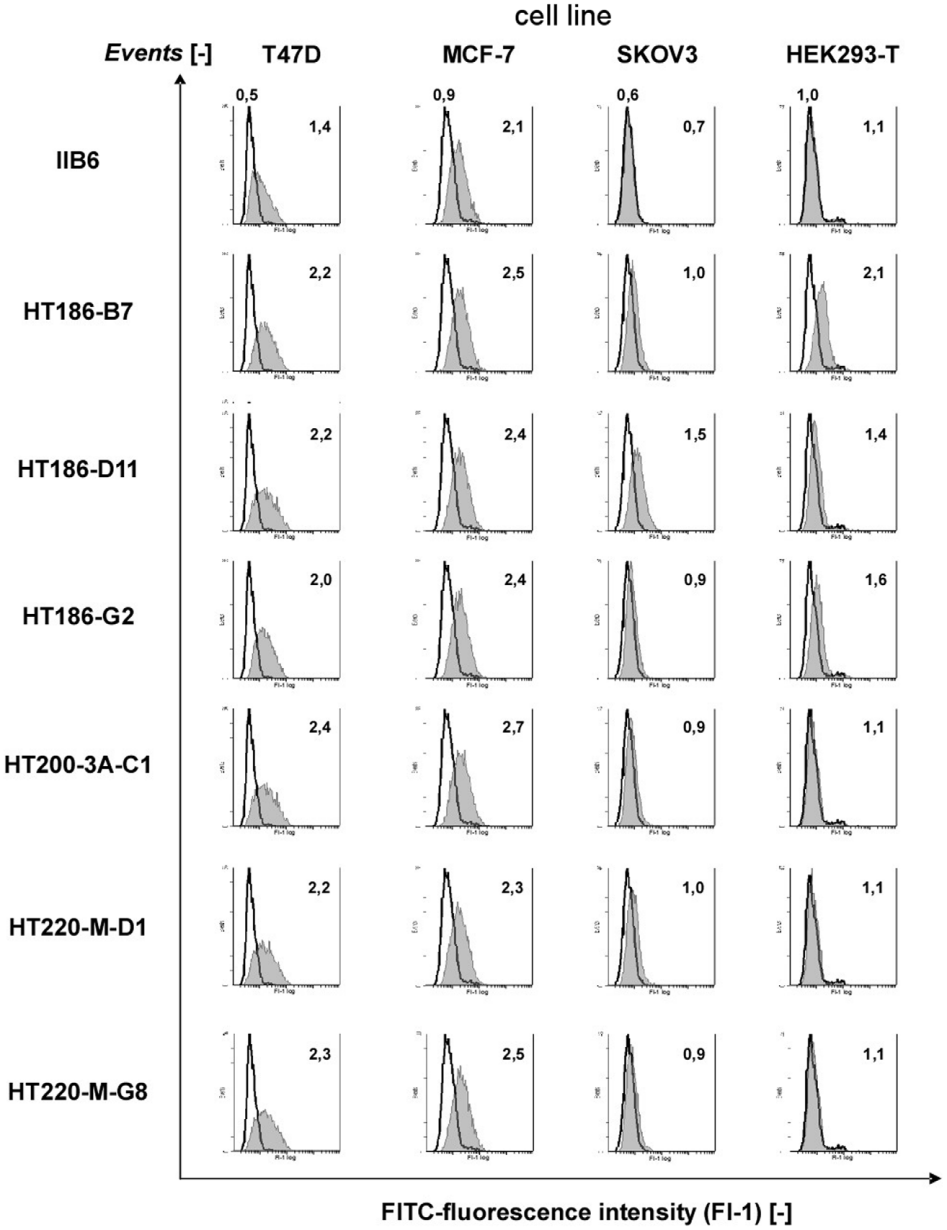
Cell stainings with purified scFv on different MUC1+ tumour cell lines and a MUC1 negative cell line (HEK293T) using FACS. 1 µg of purified scFvs were incubated on four different cell lines. The bound scFvs were detected with mouse anti-his_6_ tag IgG (1∶50) (Roche, Penzberg, Germany) and with goat anti-mouse IgG Fc specific FITC conjugate (1∶200) (Sigma). White peak: detection antibodies only, grey peak: anti-MUC1 scFv. The MFI is also given for the detection antibodies (upper row only, above the white peak), and for each scFv. 5000 cells were analysed per run.

The binding of IIB6 to native tumour associated MUC1 was analysed by flow cytometry (Fig. 3). IIB6 bound human breast cancer cell line T47D and MCF-7, but not to SKOV3. MUC1 negative HEK293T cells were not bound by IIB6.

The affinity of IIB6 to the MUC1 glycopeptide was 2,3×10^−7^ M (data not shown) and about 3,1×10^−7^ M for the MUC1 peptide (Tab. 1), as determined by surface plasmon resonance (SPR).

**Table 1:** Affinity determination (kinetic) by SPR of anti-MUC1 scFvs to MUC1 peptide antigen.

scFv	k_a_ [1/M • s]	k_d_ [1/s]	R_max_ (theor.)	K_D_ [M]	fold increase with respect to IIB6	X^2^
IIB6*	-	-	41	3.1 • 10^-7^	-	1.2
HT186-B7	3.5 • 10^4^	2.1 • 10^−5^	519	1.3 • 10^−9^	238×	7.0
HT186-D11	3.8 • 10^4^	7.2 • 10^−5^	519	5.7 • 10^−10^	544×	4.8
HT186-G2	7.2 • 10^4^	1.0 • 10^−5^	512	1.0 • 10^−9^	310×	36.7
HT200-3A-C1	4.9 • 10^4^	1.0 • 10^−4^	211	2.1 • 10^−9^	148×	4.3
HT220-M-D1	2.9 • 10^4^	1.1 • 10^−4^	200	3.7 • 10^−9^	84×	2.3
HT220-M-G8	5.0 • 10^4^	2.2 • 10^−4^	80	4.4 • 10^−9^	70×	1.5

*Kinetic evaluation was not possible for IIB6, steady-state model was used instead.

**Figure 4:**
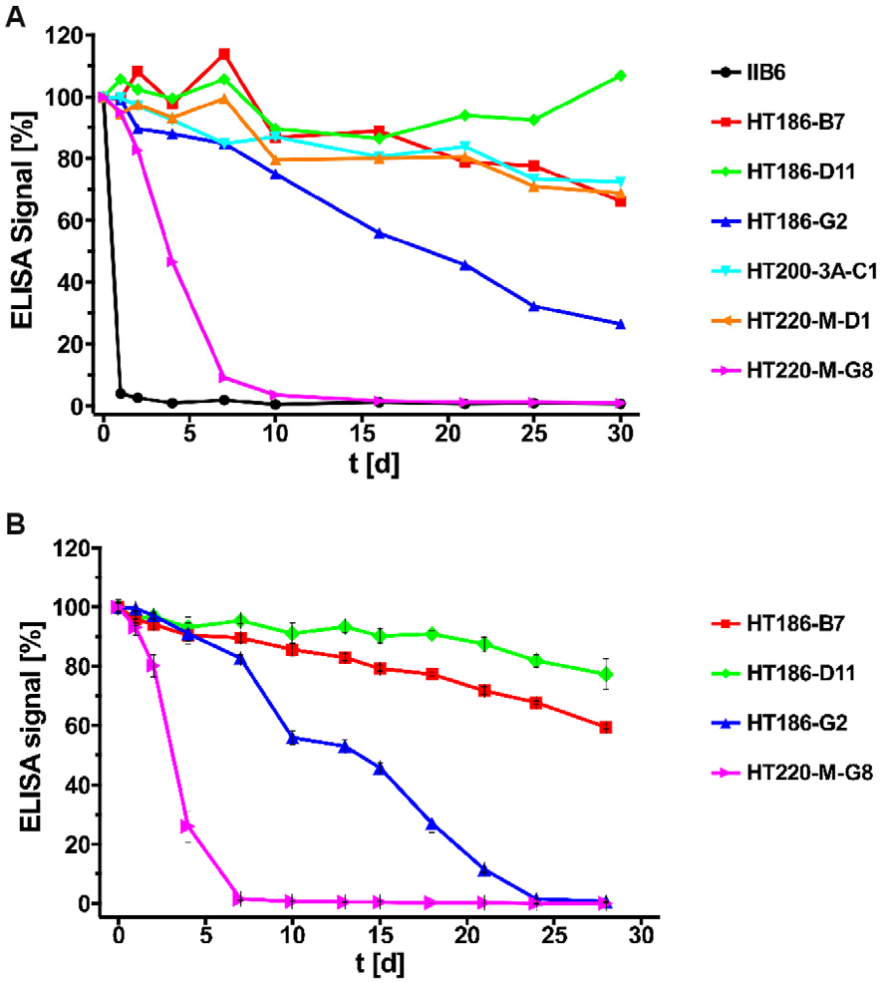
Stability analysis of the anti-MUC1 scFvs. **A** Purified scFvs (5 µg mL^−1^) were incubated in PBS up to 30 days at 37°C. After different incubation times, binding to MUC1 peptide was tested by ELISA. For each scFv, the absolute ELISA signal at t = 0 was set as 100%, all other signals refer to this value. Each well was coated with 50 µg MUC1 peptide antigen (32mer cys). Detection was performed using a mouse-anti-c-myc-IgG (9E10) (1∶500) and a goat-anti-mouse-IgG (Fab spec.) HRP conjugate (1∶10,000). **B** The same stability assay was performed with human serum instead of PBS.

However, the stability of scFv IIB6 was very low, with a half-life in serum below one day at 37°C (Fig. 4A).

### Immunohistochemistry

**Figure 5:**
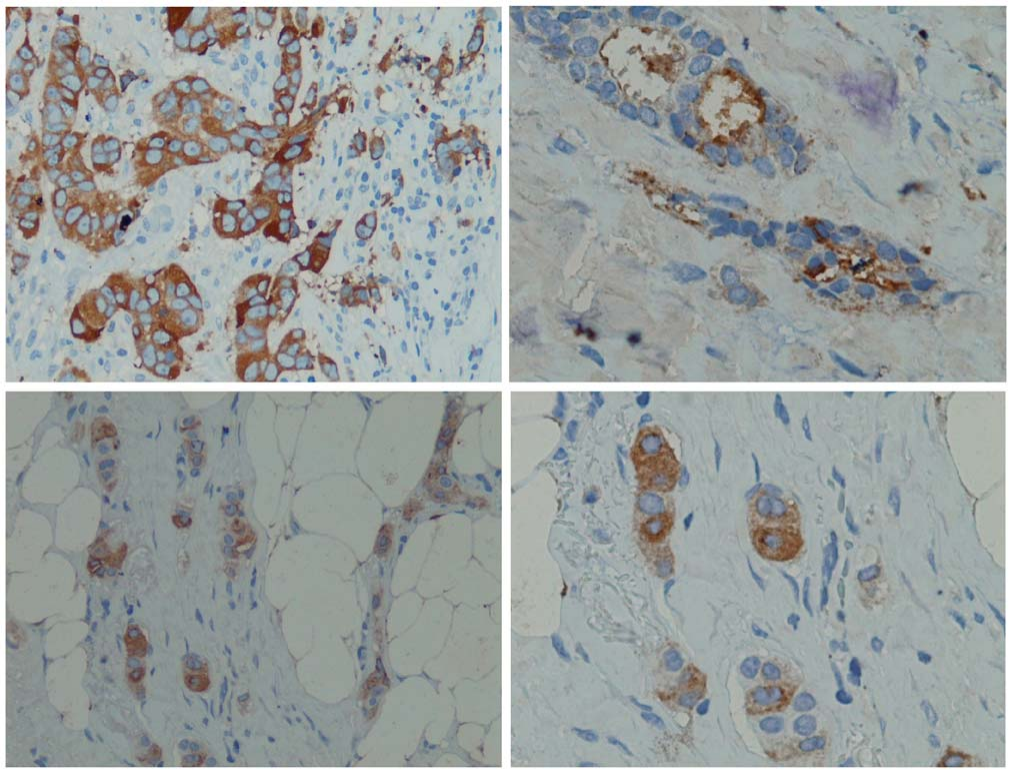
Breast cancer tissue immunohistochemistry stainings. MUC1 was stained with IIB6 (5 µg/ml), mouse anti-his (1∶1000) (Qiagen, Hilden), rabbit-anti-mouse antibody (1∶250) and detected by Peroxidase complex after Thyramine amplification (1∶200).

A panel of 228 breast cancer tissues with IIB6 was analysed by immunohistochemistry (IHC). Tumour specific staining (Fig. 5) was observed in about 80% of the different tissue samples (Tab. 2), whereas an analysis of 272 non-tumour tissue sections revealed a very low reactivity with normal tissue (Tab. 3).

**Table 2:** Analysis of the IHC stainings with mamma carcinom tissues.

intensity of staining	total frequency	percentage
negative	40	17,5%
membrane low	5	2,2%
membrane medium	18	7,9%
membrane high	9	3,9%
plasma low	43	18,9%
plasma medium	59	25,9%
plasma high	12	5,3%
both medium	15	6,6%
both high	27	11,8%
total	228	100,0%

**Table 3:** Analysis of the IHC staining with non tumour tissues.

tissue	positive staining	negative staining	comments
adipose tissue	0	8	
adrenal gland cortex	0	8	
adrenal gland medulla	0	8	
bone marrow	0	8	
cerebellum	0	8	
cerebrum	0	8	
cervix	0	8	
colon	0	8	
duodenum*	3	5	
endometrium	4	4	gland eptihel, cytogenic stroma negativ
gall bladder	0	8	
heart	0	8	
ileum	0	8	
jejunum	0	8	
kidney cortex	0	8	
kidney medulla	0	8	
liver	0	8	
lung	0	8	
lymph knots	0	8	
myometrium	0	8	
oesophagus squam. epithelium	0	8	
ovar	0	8	
pancreas	2	6	few small cell clusters (>5%)
paranasal sinus	4	4	weak, cytoplasma, in gland epithelium
parathyroid	8	0	diffuse or single cell
parotis	8	0	gland aercini negativ, only intercalated duct
placenta	1	7	synzytiotrophoblasts positiv
prostata	0	8	
sceletal muscles	0	8	
smooth muscles	0	8	
spleen	0	8	
stomach	0	8	
synovia	0	8	
testicles	0	8	
thymus	0	8	
thyroid gland*	2	6	single cells
tonsils	0	8	few sinus histocytes
ureter	0	8	
total	32	272	= 304 tissue slides

Eight slides of each tissue type were analysed.

*suspected reaction of the detection system with endogenous biotin.

ScFv IIB6 allowed a good discrimination of non-tumour versus tumour cells in a large panel of breast cancer tumour tissues, but it had a low affinity and stability. Therefore, we decided to perform an affinity maturation of IIB6.

### Affinity maturation of the IIB6

For the construction of two affinity maturation antibody gene libraries, the DNA encoding the anti-MUC1 binder IIB6 was amplified four times by error-prone PCR to introduce random mutations. The resulting IIB6 library “A” was cloned into the phage display vector pHAL14 [11], [8], [59], [60]. Three off-rate selective pannings using 1 ng, 10 ng or 100 ng MUC1/well were performed. After three weeks, 29, 74 and 81 phage particles were eluted, respectively. 92 clones were analysed by ELISA on MUC1 resulting in the identification of 28 strong binders (data not shown). All 28 binders showed different sequence mutations. Five binders with the strongest signal in the ELISA were further analysed (HT186-B7, -D2, -D11, -E8, -G2). A second library “B” was cloned by nested error-prone PCR using IIB6 to increase the amount of mutations. Here, four panning rounds in solution, followed by a pulldown with streptavidin beads, were performed. 184 clones from the third and fourth panning round were analysed as described above (data not shown). Only binders with an high ELISA signal were sequenced, resulting in three unique binders which were further analysed (HT200-3A-C1, -3A-E2, -3B-E10). The third panning round of the panning in solution was repeated and a 1000× fold excess of non-biotinylated MUC1 or the binder HT186-D11 from library “A” was used for competition. The competition was performed for seven days at 4°C. Here, 92 clones of each competition method were analysed by antigen ELISA (data not shown). About three times more binders were isolated from the panning when using the antigen MUC1 and competition with HT186-D11. The sequencing of the best binders resulted in five unique clones which were further analysed (HT220-M-C6, -M-D1, -M-G8, -D-G9, D-H11). An overview about the antibody gene libraries generated is given in table 4 and the selected affinity maturated binders are given in table 5.

**Table 4:** Overview about the constructed IIB6 mutation libraries.

Library	sequential error prone PCRs	Trans-formations	theoretical complexicity	% full size inserts	avg. point mutation rate per scFv gene
A	4	1	7.4×10^7^	45	≈20
B	7	4	1.8×10^8^	25	≈30

**Table 5:** Single antibody clones selected by three different panning methods for further biochemical charactisation.

Clone	Panning method	Competitor
HT186-B7	off rate	-
HT186-D2	off rate	-
HT186-D11	off rate	-
HT186-D8	off rate	-
HT186-G2	off rate	-
HT200-3A-C1	in solution	-
HT200-3A-E2	in solution	-
HT200-3B-E10	in solution	-
HT220-M-C6	in solution + competition	1000× excess MUC1 peptide
HT220-M-D1	in solution + competition	1000× excess MUC1 peptide
HT220-M-G8	in solution + competition	1000× excess MUC1 peptide
HT220-D-G9	in solution + competition	1000× excess soluble HT186-D11 scFv
HT220-D-H11	in solution + competition	1000× excess soluble HT186-D11 scFv

### Ranking of the antibody variants

**Figure 6:**
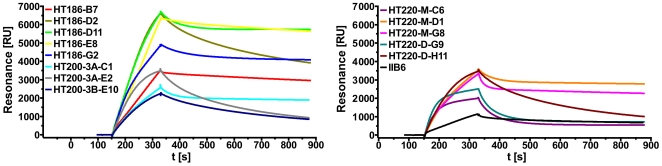
Rating of the affinity matured anti-MUC1 scFvs. Surface plasmon resonance using 90 µL of 1/10 diluted culture supernatants of the scFvs, injected with a flow rate of 30 µL/min.

To rank the affinity matured binders, the new binders and the original scFv IIB6 were produced in MTPs and the supernatant was directly used for SPR (Fig. 6). This ranking combined productivity and affinity of the binders. The association rates revealed a faster antigen-antibody interaction of all affinity matured binders when compared to IIB6. The scFvs HT186-B7, -D11, -E8, -G2, HT200-3A-C1, HT220-M-D1, -M-G8 showed much slower antigen-antibody dissociation rates. These binders were chosen for further analysis with binder HT186-E8 being excluded due to some unspecific cell binding determined by FACS (data not shown).

**Figure 7:**
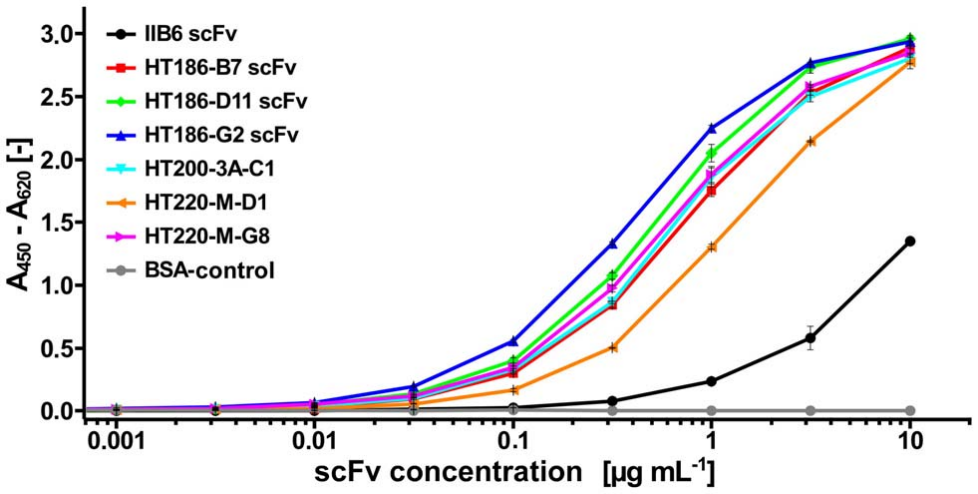
Ranking of the affinity matured anti-MUC1 scFs by antigen titration ELISA. A dilution series of scFvs was used for MUC1 detection. The bound scFvs were detected with mouse anti-myc (1∶1,000) and goat anti-mouse IgG (Fab specific) HRP conjugate (1∶10,000) (Sigma, München, Germany).

In a next step the binders were ranked by an antigen titration ELISA using IMAC purifed scFv preparations (Fig. 7). Each of the analysed affinity matured scFvs showed an increased binding when compared to the original scFv IIB6.

### Determination of affinities by SPR

The affinities of the six scFvs which bound best to MUC1 in the initial rating and IIB6 were determined by surface plasmon resonance. The results are summarized in table 1. The affinities of the affinity matured scFvs were between KD 10^−9^ - 10^−10^ M. The scFv with the highest affinity, HT186-D11, showed a 500 fold affinity increase compared to IIB6.

### Epitope mapping

**Figure 8:**
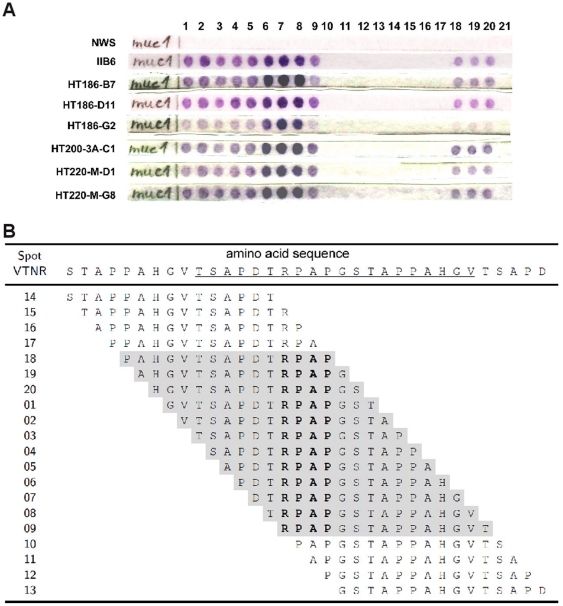
Epitope mapping. **A** The epitope mapping membrane (15mer oligopeptide, 1 amino acid overlap) was stained with 30 µg scFvs. The bound scFvs were detected with mouse anti-c-myc IgG (9E10) (1∶500) and a goat anti-mouse IgG (Fab spec.) AP conjugate (1∶2000). NWS  =  detection antibodies only. **B** Sequence overview. Amino acid sequences of the single spots on the nitrocellulose membrane. Immunostained spots were marked in grey. Amino acids forming the minimal epitope are given in bold. The sequence of one complete VNTR repetitive region is underlined.

The epitopes of the affinity matured binders were mapped by peptide spot analysis to ensure that no epitope shift compared to the original IIB6 occured (Fig. 8A, B). All binders, including IIB6, bound the same four amino acid epitope RPAP.

### Analysis of the dimerisation of the anti-MUC1 scFvs

**Figure 9:**
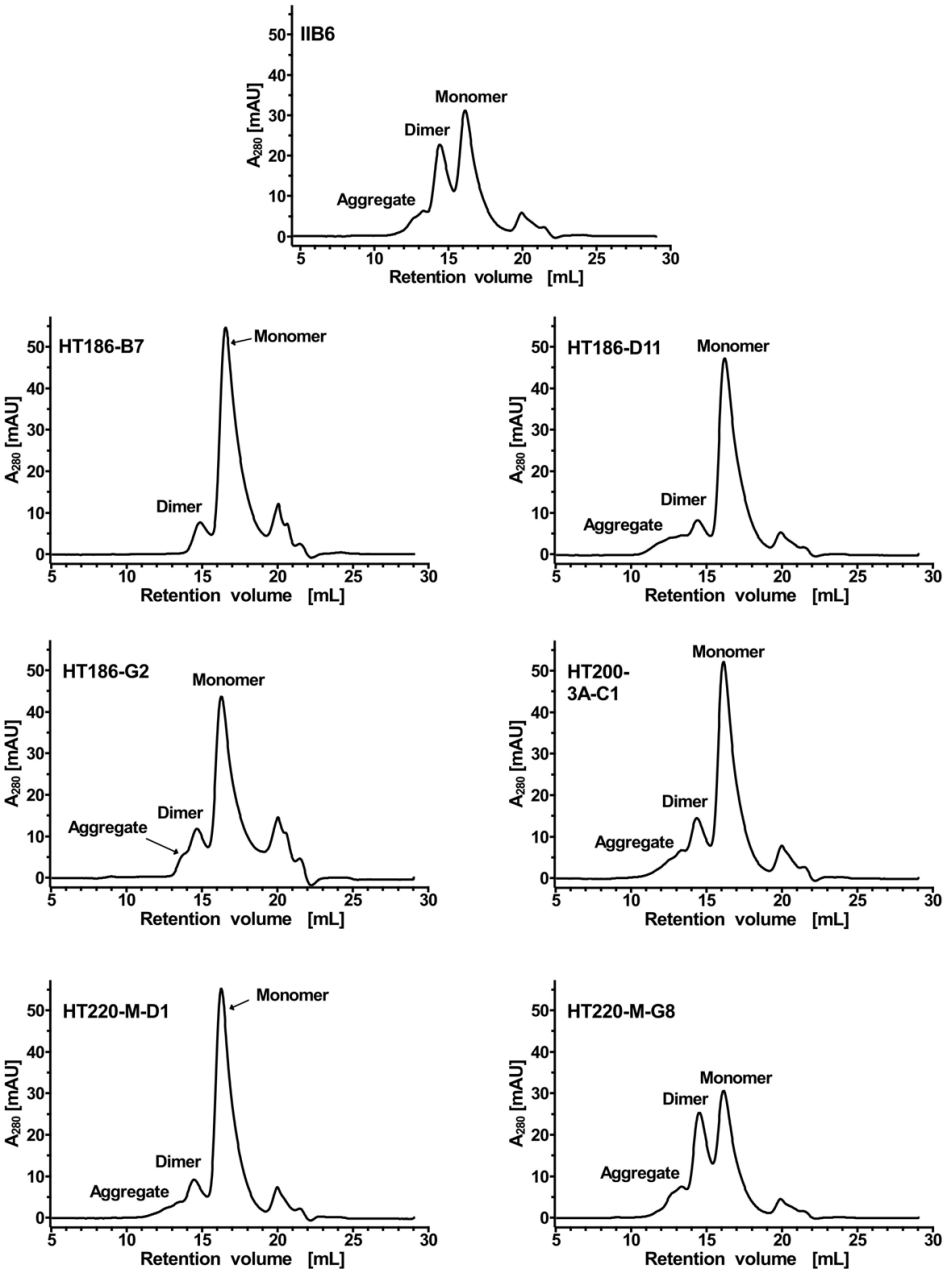
Size exclusion chromatography analysis to analyse the dimerisation tendency of the anti-MUC1 scFvs. 80 µg purified scFv fragments were separated on a Superdex200 10/300 column using PBS as running buffer with a flow rate of 0.5 mL min^−1^. The UV-absorption (A280) was drawn against the retention volume.

To evaluate the tendency of the scFv fragments to form dimers, immobilized metal ion affinity chromatography (IMAC) purified scFvs were analysed by SEC (Figure 9). The original IIB6 and HT220-M-G8 showed a strong tendency to form dimers with approximately 50% of the scFvs being dimerised. The other scFvs showed a strongly reduced dimerisation tendency.

### Analysis of the stability of the anti-MUC1 scFvs

The stability of the anti-MUC1 scFvs was analysed by incubation for 30 days at 37°C in PBS (Figure 4A), followed by ELISA to determine the binding to MUC1 peptide. The stability of HT220-M-G8 was increased in comparison to IIB6, resulting in a half-life of about 5 days. HT186-G2 had a half-life of about 20 days. The half-life of HT186-B7, HT186-D11, HT200-3A-C1 and HT220-M-D1 was longer than 30 days. Therefore, the stability of IIB6 was increased from a half day to more than 30 days by the stability and affinity maturation process.

The stability of the four best binders was further analysed in serum reflecting the situation *in vivo* (Figure 4B). Here, the half-lifes of the analysed binders were slightly shorter compared to their respective half-lives in PBS.

### Determination of the binding on MUC1 positive tumour cells

The scFv binding to MUC1 positive tumour cells T47D, MCF-7, SKOV3 and to MUC1 negative HEK293T cells was analysed by FACS (Figure 3). All affinity matured variants showed an increased binding to T47D cells, which are the tumour cells used for the initial selection of IIB6. The binding to MCF-7 was only slightly increased. Only HT186-D11 was binding slightly to SKOV3 cells. HT186-B7 showed a high background on HEK293T cells, whereas HT186-D11 and -G2 showed only a slight background. Overall, the affinity matured binders showed an improved binding to MUC1 positive cells.

### Comparison of the scFv sequences

The scFv gene fragments of the improved variants were sequenced (figure 1). A hotspot of mutations was identified in VL CDR2. Numerous mutations occured in the frameworks (FR) with highest rate found in FR2 and FR3 of VL. No mutations on the polypeptide level were found in the CDR3 and FR4 of VH and CDR1 of VL. Mutations were also identified in the linker regions. The average number of mutations was 8 per scFv, with an average of 2.6 per VH, 4.1 per VL and 1.1 in the linker region. ScFv HT186-D11 had four mutations in VH and three mutations in VL.

Humanness score [61] and germinality index [62] were analysed for the variable regions of the scFvs (table 6). Both parameters were slightly reduced by the affinity maturation process.

**Table 6:** Germinality index and humanness (Z-score) for the variable region of the anti-MUC1 scFvs.

	V_H_		V_L_	
	germinality [%]	Z-score	germinality [%]	Z-score
IIB6	85.8	−0.97	98.1	0.63
HT186-B7	85.8	−0.96	93.5	0.20
HT186-D11	82.5	−1.00	95.3	0.55
HT186-G2	82.5	−1.10	96.3	0.43
HT200-3A-C1	85.8	−0.97	93.5	0.44
HT220-M-D1	83.3	−1.18	90.1	−0.02
HT220-M-G8	82.5	−1.11	95.3	0.31

Germinality index is calculated by aligning the amino acid sequence of the variable region to the next human germline sequence [62]. Z-scores were calculated using the SHAB web interface (http://www.bioinf.org.uk/abs/shab/) [61].

Analysis of the scFv sequences with NetNGlyc 1.0 Server (http://www.cbs.dtu.dk/services/NetNGlyc/) revealed one potential N-glycosylation site in the CDR2 of VL of HT200-3A-C1 and HT220-M-D1. These clones were excluded from further experiments.

### Production and comparision of IgGs and scFv-Fc fusion proteins

Three affinity matured antibodies which are stable and did not show any potential N-glycosylation sites (HT186-D11, HT186-B7, HT186-G2) were recloned as human IgG and scFv-Fc fusion proteins, produced in HEK293T cells and purified by protein A affinity chromatography.

**Figure 10:**
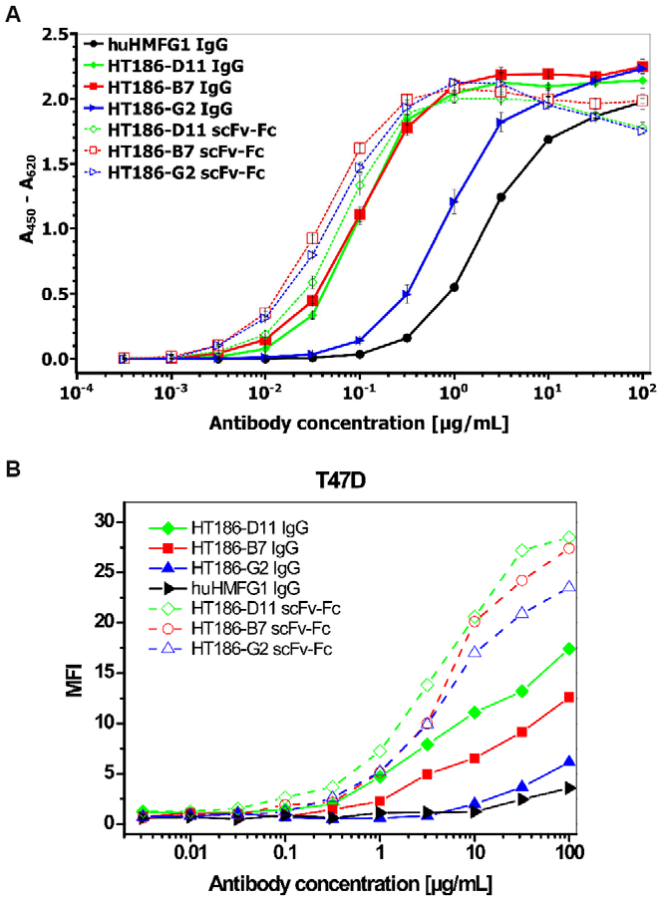
A Antigen ELISA using anti-MUC1 scFv-Fc fusion proteins and anti-MUC1 IgGs. 50 µg MUC1 peptide antigen (32mer cys) were immobilised in each well. Antibodies were incubated in different concentrations. A goat-anti-human-IgG (Fc spec.) HRP conjugate (1∶10,000) was used for detection of bound anti-MUC1 antibodies. B Flow cytometry analysis of anti-MUC1 IgG and scFv-Fc antibodies titrated on T47D. Antibodies were detected with goat anti-human IgG Alexa488 conjungate (1∶200) (Invitrogen, Darmstadt, Germany). 10000 cells were analysed per run. MFI was plotted against the antibody concentration.

These antibodies and the hHMFG1 IgG control were compared by titration ELISA using the 32 amino acid MUC1 peptide (figure 10A). The hHMFG1 showed weaker binding to MUC1 when compared to the three HT186 antibodies. The scFv-Fc variants bound slightly better than the IgG variants of HT186-D11 and HT186-B7. In case of HT186-G2, the recloning into the IgG format led to an affinity decrease of about 10fold. The binding to MUC1 positive cells was analysed by FACS (figure 10B). Here, all IgG varants bound weaker than their scFv-Fc analog with HT186-D11 showing the best binding to T47D cells, and huHMFG1 (only analysed as IgG) showing the lowest binding to MUC1.

**Figure 11:**
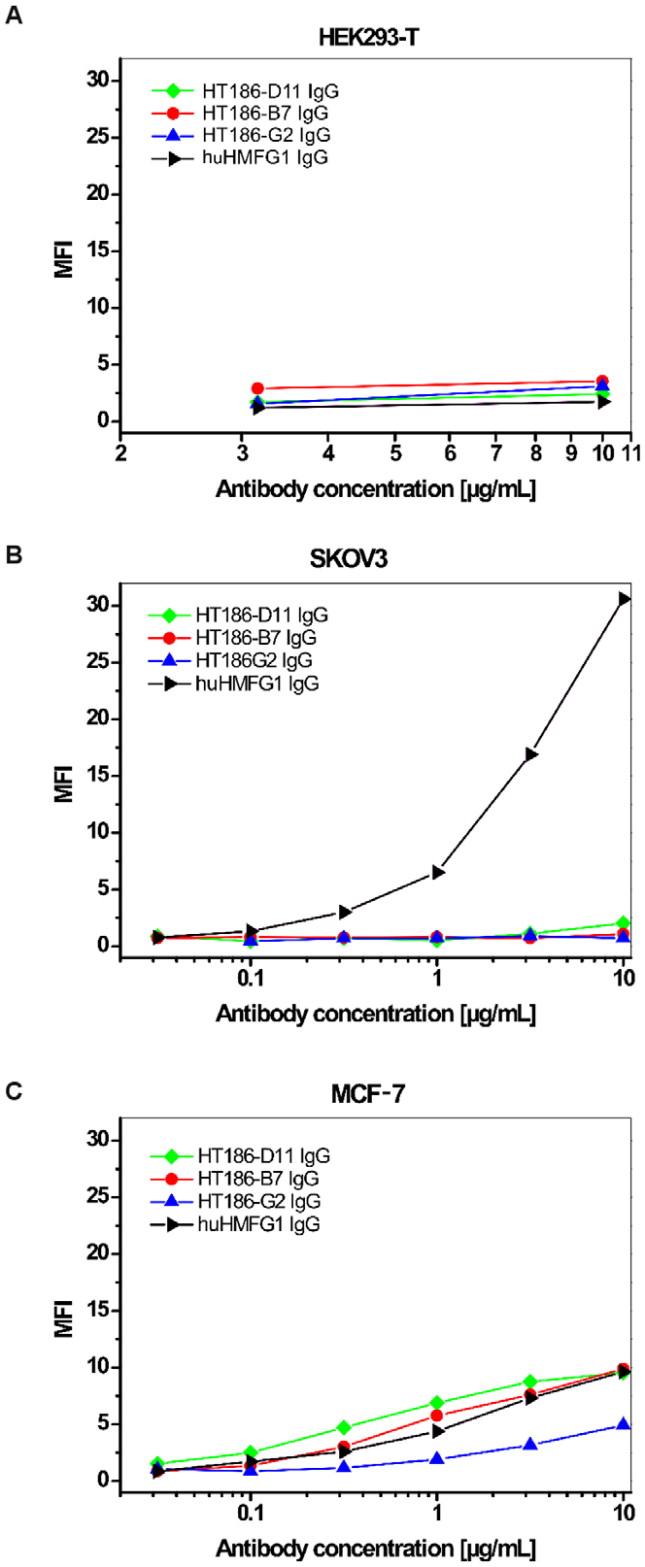
Flow cytometry analysis of anti-MUC1 IgG and scFv-Fc antibodies on different tumour cell lines HEK293T (A), SKOV3 (B) and MCF-7 (C). Different anti-MUC1 antibody concentrations were incubated on two different MUC1 positive cell lines and one MUC1 negative cell line. Detection was performed as given for figure 8B.

The IgG binding to tumour cell lines was analysed by FACS (figure 11). No binding was observed on the control cell line HEK293T (figure 11A). Only hHMFG1 bound to SKOV3 (figure 11B). HT186-D11, -B7 and hHMFG1 bound, nearly equally, to the breast cancer cell line MCF-7, only HT186-G2 showed a weaker binding (figure 11C).

### In vivo activity of HT186-D11 studied on xenograft models

**Figure 12:**
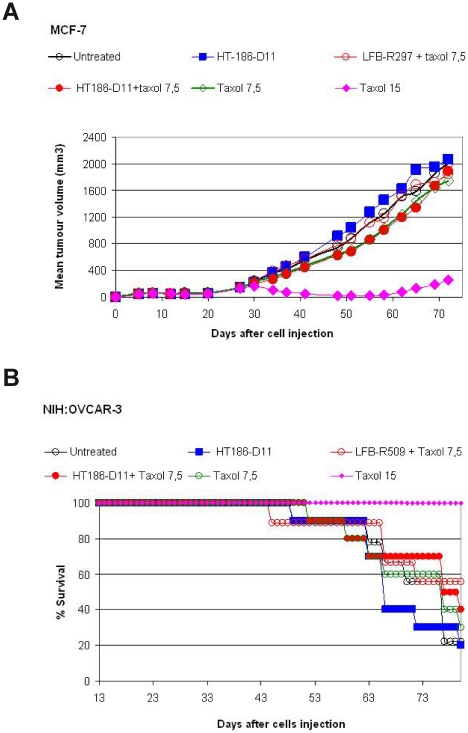
Activity studies performed in xenograft models. (A) Mean tumour volume curves observed in the MCF model. Starting on day 27, mice were IP injected with irrelevant antibody LFB-R297 or HT186-D11 at 10 mg/kg weekly and for a total of 4 weeks. These treatments were performed alone or in combination with IV injections of Taxol at 7.5 mg/kg injected once every week for 3 consecutive weeks. (B) Survival curves observed in the NIH:OVCAR-3 model. Starting on day 13, mice were IP injected with irrelevant antibody LFB-R297 or HT186-D11 at 10 mg/kg weekly and for a total of 4 weeks. These treatments were performed alone or in combination with IV injections of Taxol at 7.5 mg/kg injected once every week for 3 consecutive weeks.

The affinity matured anti-MUC1 binder HT186-D11 was analysed in MCF-7 and NIH:OVCAR-3 xenograft mouse models (figure 12). At the dose of 15 mg/kg of Taxol (paclitaxel) used as a positive control, the MCF-7 tumour cell growth was delayed in comparison to the untreated group (figure 12A). However, no tumour growth delay was observed in the HT186-D11 treated group alone or in combination with 7.5 mg/kg Taxol.

The survival rate was analysed in an intraperitoneal NIH:OVCAR-3 xenograft mouse model. Here, as previously observed in the sub cutan MCF-7 xenografts the optimal dose of 15 mg/kg Taxol resulted in an increase of survival rate in comparison to the untreated group (figure 12B). However, no survival increase was observed in the HT186-D11 treated-group alone or in combination with 7,5 mg/kg Taxol.

### ADDC

The antibodies huHMFG1, HT186-B7, -D11 and -G2 were analysed for antibody dependent cell cytotoxicity (ADCC) on MUC1 positive MCF-7 and OVCAR3 cells. Here, non of the analysed antibodies showed ADCC activity on MCF-7 cells. A slight ADCC activity was observed on OVCAR3 cells compared to the antibody independent cytotoxicity (AICC) in which huHMFG1 showed the highest ADCC, followed by HT186-G2 (data not shown).

### Internalisation assays

**Figure 13:**
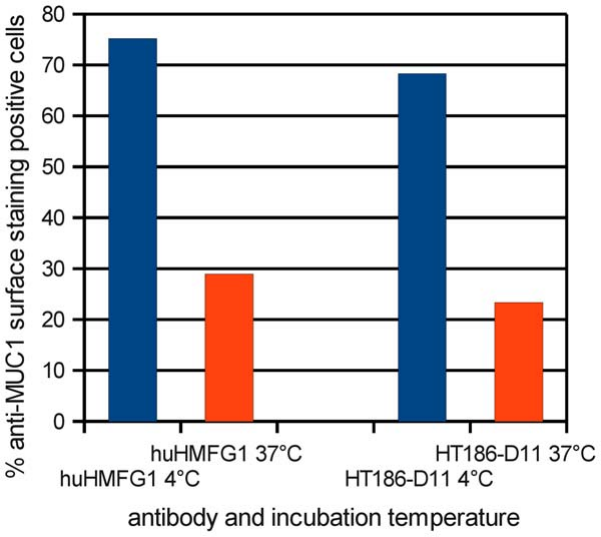
Internalisation of anti-MUC1 HT186-D11 and huHMFG1 IgGs. Internalisation was analysed by incubation of MCF-7 cells with anti-MUC1 antibodies at 37°C or on ice (on ice or 4°C, should be consistent throughout the paper) for 1h. Remaining cell surface-associated mAbs were detected by staining with PE-conjugated mouse anti-human IgG mab.

The internalisation at 4°C and 37°C for 1 h of huHMFG1 and HT186-D11 into MCF-7 cells was analysed by flow cytometry (figure 13). The percentage of MUC1 positive cells decreased from 75% (4°C) to 29% (37°C) in case of huHMFG1 and 68% to 23% for HT186-D11. Therefore, about 45% of the antibodies were internalised at 37°C according to the percentage of MUC1 positive surface stained cells.

## Discussion

MUC1 is a promising target for breast cancer tumour therapy since it is overexpressed and underglycosylated on 90% of breast cancer and also on other cancer types [21], [52].

In this work, an anti-MUC1 binder was isolated from an immune antibody phage display library constructed from MUC1 peptide vaccinated patients, a strategy which has been tested for different MUC1 positive cancers [63]–[65]. For this project, immune antibody gene libraries were used instead of naive libraries because this strategy is supposed to yield binders with a higher affinity [8], [66], [60].

The IHC studies showed that 80% of the breast cancer tumour tissues are detected by scFv IIB6. This is in accordance with the IHC studies performed with humanised anti-MUC1 binder hPankoMab [52]. Only very few nonmalignant tissues were stained with scFv IIB6. A staining of normal endometrium and pancreas was also observed for the anti-MUC1 binder PH1, whereas parathyroid was not stained by PH1 [55]. This very high specificity is an interesting observation, in light of the fact that the recognised epitope comprises a stretch of just four amino acid side chains.

Although the monovalent affinity of scFv IIB6 of 3×10^−7^ M is relatively low in comparison to other scFvs derived from immune libraries, i.e. with other specificities [67], [8], [59], [60], it is higher than the affinity of MUC1 binder PH1, which has an affinity of 1.4×10^−6^ M [55]. Interestingly, the scFvs developed here showed good binding to the breast cancer cell line T47D and ovarian cancer cell line SKOV3, but no binding to the second breast cancer cell line MCF-7.

Because of the low affinity, scFv IIB6 was affinity and stability matured. Different maturation strategies are described for affinity improvements of antibody fragments. Introducing random point mutations into the antibody gene, either by error prone PCR [68] or by *E. coli* mutator strains [69] followed by a phage display based selection under stringent conditions, allows the selection for new variants bearing improved biochemical properties. In this work, two kinds of error prone PCR libraries were generated and the screening was performed as off-rate selective pannings in MTPs, in solution and in solution with competition. Improved variants were selected using all three panning methods. Despite the small number of examples, it appears that the antibodies derived from the off-rate panning showed the best anti-MUC1 binding properties in different subsequent assays.

The affinity of the affinity matured scFv HT186-D11 was increased about 500-fold compared to the initial scFv IIB6. In the literature, affinity maturations using error prone PCR or mutator strains achieved improvement from 2 to 6000 fold [69]–[71]. Other technologies successfully used for affinity maturation are random mutagenesis followed by ribosome display [72], random mutagenesis followed by yeast display [73], chain shuffling followed by phage display [74] or by a rational design-based approach [75]. To date, affinities to MUC1 in the subnanomolar range are only described for murine IgGs binding to tandem repeats of MUC1 or MUC1-presenting cells. The apparent affinites shown for PankoMab and HMFG1/huHMFG1 are in the range of 0.9-7×10^−9^ M or 0.4–1.9×10^−8^ M, respectively, depending on the cell line used for analysis [45], [76]. A monovalent and apparent affinity is given for the human anti-MUC1 binder PH1. Here, the monovalent affinity of the Fab fragment is 1.4×10^−6^ M and the corresponding apparent affinity is 8.7×10^−9^ M [55]. Hence, HT186-D11 is the human anti-MUC1 binder with the highest described affinity.

The identified Epitope RPAP is part of the hydrophilic sequence PDTRPAP, which is described to be the most important immunogenic sequence of the VNTR region of MUC1 [77], [53]. Analysis with the murine antibody C595 which also binds to RPAP showed, that the first and the last amino acid of this antigen were essential for antigen binding [78].

We found out that nearly all affinity matured binders also had a lower tendency to dimerise. The dimerisation tendency correlated with the stability of the binder. The binders with the lowest stability showed the highest dimerisation tendency. Some of the matured anti-MUC1 scFvs were stable at 37°C for four weeks, equivalent to 1–2 years storage at 4°C and about half a year at RT [79]. To our knowledge, this is the first direct observation of a correlation of the dimerisation tendency of scFvs with the stability determined by a long term storage assay. To date, dimerisation tendencies were correlated with the stability determined by differential scanning calorimetry [80] or the effects of parameters like pH, temperature or scFv concentration [81]–[83].

The three best binders were recloned into the scFv-Fc and IgG format and analysed by titration ELISA on MUC1 and FACS analysis using T47D. In the titration ELISA, two of the three scFv-Fc fusion proteins were comparable with the corresponding IgGs. The about 10fold decrease in affinity of the HT186-G2 after conversion is influenced by 8 amino acid differences in the VH and 4 amino acid differences in the VL sequence compared to HT186-D11. In the FACS analysis, all IgG variants showed a lower cell binding compared to the scFv-Fc fusion protein.

The reason for the difference of scFv-Fc and the corresponding IgG regarding the affinity could be different VH to VL angles or other issues related to the particular IIB6 framework. When converting antibodies from the scFv format to the Fab format and vice versa, the apparent affinities were maintained or decreased [84]–[86].

The cell stainings of SKOV3 and MCF-7 were also repeated with the IgGs. Here, only huHMFG1 bound to SKOV3. On MCF-7, two of three affinity matured binders and huHMFG1 bound. The binding of all antibodies on T47D cells was much stronger than on MCF-7 cells. huHMFG1 binds the epitope PDTR whereas HT186-D11 binds to RPAP. Antibodies are binding different to different MUC1 positive cell lines [55].

The *in vitro* experiments showed no significant ADCC using established MUC1 positive cell lines MCF-7 or OVCAR3. In the *in vivo* experiments no decreased tumour growth or increased mice survival rate using MCF-7 or OVCAR3 xenograft models was observed. Recently, a clinical phage 2 trial of huHMFG1 by Antisoma (http://clinicaltrials.gov/ct2/show/NCT00770354) was discontinued because “the trial would be very unlikely to give sufficiently positive efficacy findings” (http://www.antisoma.com/asm/media/press/pr2009/2009-08-07/). To reveal the reasons for the failed xenograft experiments, ADCC was analysed *in vitro* using MCF-7 and OVCAR3 target cells. Here, no significant ADCC was detected for both huHMFG1 and HT186-D11.

Since it is known that anti-MUC1 aptamers [87] and antibodies binding to different MUC1 epitopes will be internalised [55], [88], [89], [50], [51], the internalisation of huHMFG1 and HT186-D11 was analysed using MCF-7 cells. Both antibodies were internalised by this MUC1 tumour cell line which can explain the low efficacy observed *in vitro* and *in vivo*. On the other hand, the internalisation of MUC1 specific antibodies by tumour cells allows other therapeutic approaches including the delivery of toxic compounds into the tumour cells, e.g. antibody drug conjugates or immunotoxins [90]–[92]. These concepts were tested e.g. using an anti-prostate-specific membrane antigen (PSMA) antibody coupled to ricin [93], or saporin [94], an anti-HER2 antibody coupled to Pseudomonas exotoxin A [95] or an anti-human asialoglycoprotein using the same toxin [96]. The employment of heterologous toxins raises concerns of unspecific toxicity and immunogenicity [97]. Here, the fusion of human antibodies with a human RNase may overcome these issues. The so-called immuno RNase approach was already demonstrated for an anti-CD30 antibody fused to human RNase1 [98].

In conclusion, our study provides another example how phage display based in vitro evolution was able to create an antibody with superior biochemical properties, exquisite specificity on tissue, and good product properties, by reducing aggregation and dramatically improve stability in serum. However, it also emphasises that functional *in vivo* studies should be done as soon as ever possible in the development of any therapeutic lead candidate.

## Materials and Methods

### Ethics Statement

Immunisation experiments: Human blood cell RNA was prepared from diagnostic blood sample waste obtained from a study (“klinischer Heilversuch”) on MUC1 immunisation in the Woman's Clinic of the University of Heidelberg. The materials were obtained anonymously. Following the legal standards for a “klinischer Heilversuch” valid at the time of sample preparation (Oct. 2000), neither a statement of the Ethical commission nor a patient consent was required for additional experimental work on anonymised samples from waste materials.

Mouse experiments: The animal care unit is authorized by the French ministries of Agriculture and Research (Agreement No.A21231011). Animal experiments were performed according to the European ethical guidelines of animal experimentation (Principe d'éthique de l'expérimentation animale. Directive N°86/609 CEE du 24 Nov. 1986) and the English guidelines for welfare of animals in experimental neoplasia (Workman P *et al.* UKCCCR guideline. Br J Cancer 1998, 77:1–10). All procedures with animals were submitted to the Animal Care and Use Committee of Pharmacy and Medicine University (Dijon).

### Construction of an immune antibody phage display library

The immune library was constructed from peripheral blood B lymphocytes from mamma carcinoma patients which were vaccinated with a VNTR MUC1 Peptide (APDT(GalNAc)RPAPGSTAPPA). The library was constructed using the vector pSEX81 [57] and a human primer set [56] according to [99].

### Selection of anti-MUC1 binders

The panning and screening was performed according to [100]. Breast Mucin Antigen (BMA) was purified by affinity chromatography from cells of the human breast cancer cell line T47D using mouse mAb BM7. Panning rounds were performed on 10 µg MUC1 (BMA) and a MUC1 glycopeptide (APDT(GalNAC)RPAPGSTAPPA-C).

### SDS-PAGE and immunoblot

Samples of total cell lysates were run on a reducing 7.5% SDS-PAGE and electroblotted onto nitrocellulose filter. The filter was blocked for 2 h at RT in PBS, 2% skimmed milk. After washing the staining was performed using 5 µg/mL IIB6, followed by detection of the His-tag with mouse anti-(His)_5_ mAb (Qiagen, Hilden, Germany) (1∶1000) and goat-anti-mouse IgG HRP conjugated (Dianova, Hamburg, Germany) (1∶5000). The detection was performed using NovaRed Substrate Kit (Vector, Burlingame, USA).

### Immunohistochemistry

Immunohistochemical staining were done on formalin-fixed and paraffin embedded human breast samples and 304 healthy tissues from 38 different organs/locations according to [101]. All samples were retrieved from the archives of the Institute for Pathology, Medizinische Hochschule Hannover. In brief, slides were deparaffinized in xylene and rehydrated in graded alcohol. Heat induced epitope retrieval using the microwave technique (citrate buffer pH 6.0, 20 min at 100°C) was followed by blocking of endogenous peroxidase with 3% H_2_O_2_ as well as endogenous biotin by an Avidin/Biotin-blocking Kit (Vector Laboratories, Burlingame, Ca, USA).

The primary scFv IIB6 was incubated (5 µg/ml) overnight at 4°C. The detection was performed using mouse anti-his (1∶1000) (Qiagen, Hilden), followed by biotinylated secondary rabbit-anti-mouse antibody (1∶250) (Zymed Laboratories, San Francisco, CA, USA) at room temperature and detected by a sensitive Peroxidase complex after Thyramine amplification (1∶200) (NenLifeScience, Boston, MA, USA). DAB served as substrate and hematoxylin for counterstaining.

### Construction of the mutation libraries

1 ng template DNA of the initial anti-MUC1 binder IIB6 was amplified in a volume of 25 µL using a random mutagenesis PCR kit (GeneMorphII, Stratagene) and 0.2 µM of the oligonucleotide primer HT-IIB6-Aff_fwd (5′ tgctggcagctcagccggccatgg 3′) and HT-IIB6-AFF_rev (5′ tgatggtgatgatgatgagcggccgc 3′)) for 35 cycles (94°C 60 s, 65°C 60 s, 72°C 70s) followed by a 10 min final synthesis step. The PCR products were purified by agarose gel electrophoresis using the Nucleospin Extract 2 Kit (Macherey-Nagel, Düren). This step was repeated three times for library “A”. The PCR products were purifed and cloned into the phagemid pHAL14 using the restriction sites *Nco*I and *Not*I.

The library “B” was cloned in the same way as library “A” with following modifications. The random mutagenesis was performed by nested PCR using sequential three sets of primers: MHLacZ-Pro_f (5′ ggctcgtatgttgtgtgg 3′)/HT-gIII-Beginn1-rev (5′ taaacaactttcaacagtttcagct 3′), MKpelB_f (5′ gcctacggcagccgctgg 3′)/MKmyc_r (5′ gatcctcttctgagatgag 3′) and HT-IIB6-Aff_fwd/HT-IIB6-AFF_rev. In total, the random mutagenesis PCRs were performed seven times.

The libraries were packaged using M13K07 as described by [102].

### Selection (panning) of affinity matured scFvs

The off rate selection was performed as follows: MUC1 15 aa peptide with cystein (APDTRPAPGSTAPPA-C) was coated over night in different amounts (100 ng, 10 ng, 1 ng) using sodium carbonate buffer pH 9.7 (35 mM NaHCO_3_, 15 mM NaHCO_3_) into Nunc Maxisorp stripes (Nunc, Langenselbold, Germany), followed by blocking with 1% (w/v) BSA in M-PBST (phosphate buffered saline [103]+0,1% Tween 20+2% skim milk powder) for 1 h at RT. Afterwards, the wells were washed with PBST using an ELISA washer (TECAN Columbus Pro). About 4×10^10^ phage particles of the mutation library “A” were incubated into each well for 3 h in M-PBST followed by 30× stringent bottom wash using an ELISA washer. Afterwards the stripes were incubated for one week in 2 L PBS under gentle shaking at 4°C. The stripes were washed 20× as described above and incubated again for one week in 1 L PBS at 4°C and gentle shaking followed by 20× washing. Afterwards the stripes were incubated for the third week as described above. The wells were washed 3× with the standard washing protocol and the remaining scFv-phage were eluted using 200 µL 10 µg/mL trypsin at 37°C for 30 min. *Escherichia coli* XL1-Blue MRF' (Stratagene, Amsterdam) were grown up to O.D._600_ 0.4–0.5. 50 µL bacteria were infected with the eluted antibody phage, plated on 2×TY agar plates [103] +100 µg/mL ampicilin +100 mM glucose and incubated over night at 37°C.

The panning in solution was performed as follows: 50 µL streptavidin beads (Dynabeads M280, Dynal, Oslo) were incubated with M-PBST for 1 h. All incubation steps were performed in an overhead shaker. The beads were captured using a magnetic separator (Dynal MPC, Dynal, Oslo). The blocked beads were incubated with about 1×10^13^ phage particles of mutation library “B” for 30 min to deplete bead binders. The supernatant with the residual library was incubated with 60 nM biotinylated 32 aa MUC1 peptide (Biotin-βA-βA-APDTRPAPGSTAPPAHGVTSAPDTRPAPGSTA) in M-PBST with 2% BSA (bovine serum albumin) for 1 h at RT followed by an incubation with about 7×10^7^ blocked streptavidin beads for 15 min. The beads with the bound antibody phage were captured by pull down in a magnetic separator. The beads were washed 20 times with PBS. Elution of the bound scFv phage particles with trypsin and reamplification of the scFv phage were done as described for panning in MTPs by Hust et al. 2007 [104]. In total three panning rounds were performed.

The panning in solution with competion was performed as follows. The procedure was analogous to the panning in solution with the following modifications. After incubation of the mutation library with biotinylated MUC1 peptide, an excess of the competitor, 1 µM non-biotinylated MUC1 peptide or soluble MUC1 binder, was added (1000× excess) and incubated for an additional week at 4°C.

### Production of scFvs in microtitre plates (MTPs)

For the identification of monoclonal binders, colonies from the titre plates of the eluted phage particles after panning were picked and soluble scFvs were produced in microtitre plates as described before [105].

### Enzyme linked immunosorbent assay (ELISA)

For anti-MUC1 ELISA, MUC1 32 aa peptide with a C-terminal cystein (APDTRPAPGSTAPPAHGVTSAPDTRPAPGSTA-C) was coated to 96 well microtitre plates (Maxisorp, Nunc) in PBS over night at 4°C. After coating, the wells were washed three times with PBST and blocked with 2% (w/v) skim milk powder in PBST (2% M-PBST) for 1.5 h at RT, followed by three washing steps with PBST. For the antigen ELISA soluble scFvs, scFv-Fc fusion proteins or IgG were diluted in 100 µL 2% M-PBST and incubated in the MUC1 coated plates for 1.5 h at RT followed by three PBST washing cycles. Bound scFvs were detected with the murine mAb 9E10 which recognises the C-terminal c-myc tag and a goat anti-mouse serum conjugated with horseradish peroxidase (HRP) (Sigma; 1∶10,000). IgG and scFv-Fc fusion proteins were detected using goat anti-human Fc specific serum conjugated to HRP (Sigma; 1∶20.000). The visualisation was performed with TMB (3,3′,5,5′-tetramethylbenzidine) as substrate and staining reaction was stopped by adding 100 µl 1 N sulphuric acid. Absorbance at 450 nm was measured by using a SUNRISE™ microtitre plate reader (Tecan, Crailsheim, Germany).

### Surface Plasmon Resonance (SPR)

Surface plasmon resonance was performed using Biacore 2000 according to the Biacore manual. Briefly, about 50 RU recombinant MUC1 15 aa with cysteine peptide (APDTRPAPGSTAPPA-C) were coupled in 10 mM sodium acetate buffer pH 4.0 on a CM5 chip after activation with NHS/EDC chemistry, followed by PDEA in 80 mM borate buffer. For reference, 50 RU of a control peptide were coupled in 10 mM sodium acetate buffer pH 4.5. Remaining active groups were saturated with 50 mM L-cysteine. Serial dilutions of scFv (0 nM –400 nM) were measured at a flow rate of 50 µL/min. The chip was regenerated with 100 mM glycine buffer pH 2.5. Data fitting was performed using 1∶1 Langmuir separate fitting algorithm of the Biaevalution software.

### Production of scFvs in *E. coli*

For production of scFvs in *E. coli*, scFvs were recloned into the vector pOPE101-XP according to [105].

The affinity matured scFvs were produced in shake flasks according to [106] with modifications. Briefly, 300 mL 2×TY [103] +100 µg/mL glucose +100 µg/mL ampicillin were inoculated with an over night culture to O.D._600_≈0.15 and cultured at 37°C and 250 rpm. The induction was started by adjusting to 50 µM IPTG at O.D._600_ = 0.9 and the cells were cultivated at 25°C and 250 rpm for 3h. Bacteria were harvested by centrifugation for 5 min at 4200×g at RT. Pellets were resuspended in 30 mL ice cold PE buffer, pH 8 (500 mM sucrose, 100 mM Tris, 1 mM EDTA) and incubated for 20 min on ice, interrupted by short vortexing every 5 min. Subsequently the bacteria were pelleted for 30 min at 30000 xg at 4°C. The supernatant (periplasmic fraction) was stored at −20°C. The pellet was resuspended in 30 mL ice-cold dH_2_O and incubated for 20 min on ice, interrupted by short vortexing every 5 min. Spheroblasts were pelleted for 30 min at 30000×g and 4°C. The supernatant (osmotic shock preparation) was stored at −20°C.

### Epitope mapping

A series of 20 peptides (15mers overlapping by 14 amino acid residues) representing the entire VNTR sequence of human MUC1 were synthesized as an array on amino-cellulose membrane by SPOT-synthesis [107], [108]. This membrane-bound peptide array was probed with scFv antibody fragments as described above for the immunoblot except that MTT/BCIP [100 µL 1 M MgCl_2_; 80 µL BCIP (15 g/L in DMF); 120 µL MTT [3-(4,5-dimethylthiazolyl-2)-2,5-diphenyltetrazoliumbromide] (50g/L in 70% DMF +30% H_2_O_2_) in 20 mL CBS (8 g/L NaCl, 0.2 g/L KCl, 2.08 g/L citrate, pH 7.0)] was used for staining.

### Size exclusion chromatography

Size exclusion chromatography was performed using Superdex200 10/300 GL column (GE Healthcare) on an ÄKTA purifier system (GE Healthcare). 100 mL of each sample were injected and separated by using PBS as running buffer with a flow rate of 0.5 mL/min. A280 was plotted against the retention volume to identify the different antibody fragments, in comparison to a set of marker proteins analysed in separate runs.

### Stability assay

The stability assay was performed according to [60]. Briefly, scFvs were aliquoted as triplicates in 100 µL (5 µg/mL in PBS or human serum) in 2 mL microtubes (Sarstedt, Nürnbrecht, Germany) and stored at −80°C. Every three days and every day for the last two samples, an aliquot was thawed and transferred into a 37°C incubator. All samples were analysed at the same day in the same antigen ELISA using MUC1 32mer Cys peptide as antigen bound to microtitre plates (Nunc). A total of 80 µL of the scFv solutions was analysed by antigen ELISA.

### Flow cytometry

Tumour cells were cultured on poly-L-lysine coated 10 cm diameter tissue culture plates at 37°C and 7% CO_2_ using media specific for the cell lines. The cells were harvested at 70% confluence. Therefore, the cells were washed with 10 mL PBS and afterwards detached using 1 mL trypsin solution (10 mg/L trypsin in PBS). The cells were centrifuged at 1000×g for 5 min and resupended in 3 mL FACS buffer (2% (v/v) fetal calf serum, 2 mM EDTA in PBS) with the anti-MUC1 scFv, scFv-Fc or IgG on ice for 1 h. The cells were centrifuged as above, resupended in 3 mL FACS buffer, centrifuged again in resuspended again in FACS buffer with mouse anti-his6 tag (1∶50) (Roche, Penzberg, Germany) for scFv staining or goat anti-human IgG Alexa488 conjugated (1∶200) (Invitrogen) for scFv-Fc or IgG staining on ice for 1 h. For scFv staining the cells were washed again and resuspended in FACS buffer with goat anti-mouse IgG FITC conjugated (1∶200) (Sigma, München) on ice for 1h. Finaly, cells were washed, resuspended in 500 µL FACS buffer and analysed by flow cytometry using a FC500 with two lasers (488 nm and 633 nm, Beckman Coulter, Germany). For each sample 5000 (scFv) or 10000 (scFv-Fc, IgG) events were measured and data were analysed using CXP analysis software (Beckman Coulter).

### Production of IgG and scFv-Fc fusion proteins in mammalian cells

The scFvs were recloned into the scFv-Fc format using the vector pCMX2.5-hIgG1-Fc-XP according to [5].

Genes encoding VH and VL of hHMFG1, HT186-B7, -D11 and -G2 were orderd as synthetic genes with codon optimisation for rat (Genscript, Piscataway, NJ, USA). The mammalian expression vector CHK622-08 was modified by replacing the C kappa with C lambda derived from the vector CHL558-0. VH was cloned between *Apa*I and *Nhe*I and VL was cloned between *Spe*I and *Dra*III. All vector constructs were checked by DNA sequencing.

### Xenograft experiments

Antitumour activity of HT186-D11 was evaluated on two xenograft models. The models consisted of female Balb/c *Nude* mice subcutaneously injected with the MCF-7 mammary human tumour cell line or intraperitoneally injected with the NIH:OVCAR-3 ovary human tumour cell line. In the latter model, the cell line was amplified *in vivo* in SCID mice and ascites were collected and injected into Balb/c *Nude* mice.

Mice were treated weekly with 10 mg/kg of antibody for a total of 4 weeks injected by the intraperitoneal route. Antibodies recognising human CD20 (LFB-R509) and D (LFB-R297) antigens were used as controls. Treatment started on day 27 in the MCF-7 model and on day 13 in the NIH-OVCAR-3 model. Antibodies were administered alone or in combination with Taxol (paclitaxel) at 7.5 mg/kg weekly for a total of 3 weeks and injected by the intravenous route. Two groups treated with Taxol only administered at 7.5 and 15 mg/kg were used as a control for the chemotherapy treatment. Taxol treatment was administered 1 day after the antibodies.

Antitumour efficacy was evaluated in the MCF-7 model by measurement of the tumour volume and in the NIH:OVCAR-3 model by following the survival. The tumour volume was calculated with the following formula where length corresponds to the largest tumour diameter and width to the smallest tumour diameter: TV = (length × width^2^)/2.

### ADCC

The antibody-dependent cell-mediated cytotoxicity assays (ADCC) were performed using purified human NK cells as effector cells. Human NK cells were purified from the peripheral blood of healthy donors by the negative depletion technique developed by Miltenyi Biotec (Bergisch Gladbach, Germany).

The target cells, MCF-7 and OVCAR3, were mixed with NK cells at an effector-target (E/T) ratio of 20/1 in the presence of antibody dilutions. After 16 hours of incubation at 37°C and 7% CO_2_, cytotoxicity was quantified using the cytotoxicity Detection Kit (Roche Applied Sciences) based on lactate dehydrogenase (LDH) released into the supernatants. Data was expressed as the percent of lysis calculated according to the following formula:
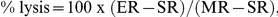
where ER, SR, and MR represent experimental, spontaneous, and maximum release, respectively. ADCC values were expressed as:
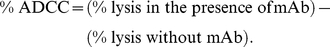



### Internalisation Assay

To address the possibility that incubation at 37°C of anti-MUC-1 mAb-labeled MUC-1+ cells results in mAb internalization, the MCF-7 cells (2×10^5^ cells) were incubated with 10 µg/ml of anti-MUC1 antibodies (huHMFG1 or HT186-D11) on ice for 1h. After washing in cold PBS, 2% FCS, cells were separated in two groups and incubated for 1 h at 4°C and 37°C, respectively. After washing, the remaining cell surface-associated mAb was detected by staining with PE-conjugated mouse anti-human IgG mab (Beckman Coulter).
